# Art and Science in Medicine

**Published:** 1983-04

**Authors:** H. K. Bourns

**Affiliations:** Consultant Surgeon Emeritus, United Bristol Hospitals


### Art and Science in Medicine

**H. K. Bourns, B.A., M.B., B.Ch., F.R.C.S.**
Consultant Surgeon Emeritus, United Bristol Hospitals

The Bristol Royal Infirmary was opened in December 1737,[1] and the 'other hospital' was founded and opened on November 1st, 1832.[2]

There was some healthy and unhealthy rivalry, and a pamphlet entitled 'A Rational Appeal to Humanity and Benevolence relative to the Bristol General Hospital'[3] was written by Abraham Bignell, M.D., and widely circulated at that time. It was largely an attack on Dr. E. Long Fox, senior, who had tried 'Mesmerism' on some of his patients after a French doctor call Mesmer, and a condemnation of the Stethescope which he described as 'horrible quackery'.

As I considered all this I realised that the medical profession and those who dealt with people have often been in opposition, and it would seem as if all the scientific advances (and there are many) have not been able to defeat the unorthodox in the care of the sick. The medical profession must, however, have an inbuilt and fundamental motivation to help those who are finding life hard and difficult because of disease.

In July 1883 the first number of the Bristol Medico-Chirurgical Journal was issued under the editorship of Greig-Smith,[4] who was elected to the staff on June 20th 1876. J. W. Arrowsmith did the printing and the publishing.

Mr. Arnold Aldis, Surgeon on the Professorial Unit in Cardiff until very recently, wrote about the Doctor's 'Responsibility for the Society we Serve' on the 29th January 1970.[5] 'As medical practitioners in the widest sense we are accustomed to the ideal of service as a strong motivation in the profession. In the past this concept has been maintained chiefly on a personal level and devoted to patients as individuals. Such personal service, I trust, still remains a prominent and guiding principle in the ethos of medicine'.

In general practice the single handed practitioner has almost completely disappeared, giving way to the Health Centre and the Group Practice serving a much larger population.

As the influence of the Christian Church was declining, many people turned to the medical profession, and medicine had a very wide commitment thrust upon it.

Presidential Address given to the Bristol Medico-Chirurgical Society, October 1982.

If the generality of the public have neglected the standards and influence of the Church, has the profession anything to offer? Is it the responsibility of the profession to take on the mantle of moral leadership? No matter what attitude the people adopt to the moral leadership of the Church, leadership is necessary, and if the professions such as medicine and law will not take on the role of leaders of society, then the nation can be led down rather than up, and positive moral leadership is vital for a positive caring community.

However, we must not be lost in the realm of national and community considerations because the individual is the basis of society, and the medical profession has a unique opportunity to take on leadership and thus to influence society, but only by positive approaches to the health of the individual in the widest sense. The doctor has the power to influence society through his influence on the individual, and if the profession does not accept this challenge there are plenty who will.

Fringe medicine is now propounded by the profession, and much thought is being given to the various groups who are available for the care of patients who have found the doctor wanting, and the available therapy to be unsatisfying.

The patient has a long tradition of guiding principles which have influenced individuals and propped them up in the difficult as well as in the prosperous and stable times. If one looks back through time one finds that men and women have turned to many forms of leadership as individuals and as a community, so that modern scientific medicine has to fill a role for individuals as well as for the community. The individual and his or her relations and their relationships must remain the basis of positive living, and unless the medical profession comes alongside the problems of society others will take its place because the individual will seek 'others' if the orthodox practitioner will not get involved.

A scientific approach with advances in medicine is the standard, and as long as science is advancing medical care must be improving, but the ungrateful nation will not accept the fact that all is well. Science cannot take over the role of all the caring influences that the public have used and by which they have been exploited in the past. Medicine is an art not a science only, and the patients' knowledge of advances in medical care can only be learnt from individual patient to doctor communications in spite of the journalists and broadcasters – many of them medical – who seek to spread the facts or the psuedo-scientific facts, many of which are made to appear very exciting.

In Health Trends in February 1982[6] Sir Douglas Black discussed the aims of the Health Service. His principles are –

(1) To relieve established and perceived illness by cure if possible, but at least by palliation of the symptoms.

(2) To prevent disease both by promoting a healthy lifestyle and by specific interventions such as immunisation.

(3) To provide a framework in which health service workers can attain fulfilment at work.

Is the Health Service at the cross roads, and has it been so for some time? Professor George Pickering in 1977 posed the question 'Medicine at the cross roads – a learned profession or a technological Trade Union?'.[7]

This may seem to be far fetched to many people because they will not allow themselves to think of unrest in such clear-cut terms. This may be true, but there is no doubt that the separate agitations of the various groups in the Health Service have not necessarily led to an improvement in the treatment of patients. The changes that take place must lead to either improvement or deterioration because major upheavals lead to major results. The changes in conditions as far as staff are concerned may indirectly affect relationships with patients if the new environment of the life of the health worker is so much better that they are more dedicated and less worried people. The present political unrest is a disaster no matter what the outcome, because a caring community cannot but lose some of its motivation as far as healing is concerned.

Doctors in general, and senior doctors in particular, have been rather disinclined to consider it to be any way part of their function to be concerned about the conditions of work of the nurses and other members of the Health Service staff. Maybe the present division of attitudes in the Health Service will lead to disruption of routines and lack of co-operation with an inevitable reduction in patient care, or there may be a coming together because the recognition of each member of the health team has been brought to everyone's notice.

The two great functions of the N.H.S. must be firstly the treatment of disease and secondly the prevention of disease.

The exciting part of the N.H.S. is the treatment of disease. No matter what the medical student regards as his objective when he embarks on his course of training, I think that there are two main reasons for chosing medicine as a career.

Firstly, many senior school children have been brought up in scientific subjects because they were allowed to abandon classics and languages at the 'O' level stage. What does science lead to? There are many answers to that question, but medicine offers the possibility of a career as a scientist which is satisfying and is likely to lead to a well rewarded life both emotionally and financially.

Secondly, some of the medical students and especially the parents, have a romantic view of 'the doctor'. He is a God-like person to whom one turns when in trouble, and if one has had contact with a G.P. who is a personal friend, then the idea of reaching that goal is likely to have a powerful attraction and pull.

Thirdly, family connections are important.

Thus, their motivation may be either too romantic or too scientific, so that they may become disillusioned both during training and after qualification. Medicine is satisfying, but there are many emotional and practical adjustments that have to be made in order to bring the true and false images together. The vision and the reality may indeed be far removed at the outset of a medical career, and life does not give anything away. A medical career is a hard taskmaster, and the initial enthusiasm must be superceded by a dedication and primary objective which will carry one through the hard life circumstances which may appear from time to time.

A Health Service is an open-ended commitment. It therefore tends to become a 'National Ill Health Service'. An ill patient is bound to take time, energy and finance. All these factors are limited within the country as a whole. Should we not recognise all the agencies that are available to people. 'From the cradle to the grave' is the slogan of the Welfare State. A vast majority of our citizens however, have access to many types of service or 'second opinion' in the course of a normal way of life. Some of these are provided by the Health Service in both the national and Local Authority services. Baby clinics, welfare clinics, school clinics, industrial services and health visitors and clinics are available during a normal life so that it could be argued that the average citizen has a double service available.

What does more and more State intervention mean? In these days of small families and careers for all, the care of an independent person is a problem. It is a problem for either an individual or the State, or both. If the State, then this is another open-ended commitment over which the planners have no control. Whatever Service is taken over leads to the necessity for more and more expansion and expenditure. As there is no direct financing the relative has to pay for the service either by rates or taxes. We cannot go on allowing the public to believe that all this is 'free', because the bias towards the State Service rather than an independent service is based on this false assumption.

There is a double service for most citizens, and if they subscribe to an independent service paid for by regular subscription to an Insurance Agency, then some people have a threefold service available.

The N.H.S. suffers from the fact that there was a belief that after the service had been going for a few years the burden of illness and the cost of coping with it would fall to a lower level. This has not happened, and preventive measures based on scientific methods found during research does not seem to have helped towards national economies. It could be argued that the situation would have been much worse if the preventive measures taken had not been introduced.

Clinical medicine does have a preventive element which is not unimportant. The rapid cure of an acute illness may in many cases prevent the development of chronic ill health. At one stage and in many cases it could be said that the difference between no medicine and good medicine was small, but in specific cases improvement leads to a definite speeding up of the healing process and the prevention of complications.

In 1976 Thomas McKeown[8] analysed the effect of medicine over the years and showed without any doubt that much of the improvement in the morbidity and mortality of disease was due to changes in environment and nutrition. Since 1700 when records were available, there was a steady decline in death rates, and in particular since 1841 when registrar general figures became known.

The problem of comparison in the disease process is handicapped by diagnosis. Infections are undoubtedly a potent factor, and it was not until 1935 that medicine could claim a dramatic change when sulphonamides and later antibiotics were introduced. Before that immunisation, active usually, was a factor but not a very great one in reduction of disease and the death rate.

Most diseases, including the common ones, are not inevitable. They result from environmental influences or genetic material which is varied, complex, and at present little understood.

This is in accord with past experience. The improvement in health since the 18th century was in respect of post-natal rather than pre-natal conditions. The advances in health have come with better nutrition, better hygiene and later birth control.

Personal interest in positive health would be a healthier approach. We have a National Ill Health Service, and what is required is a Natural Health Service. Health measures are in the control of the individual and there are signs that habits such as smoking, eating, exercise and the like are being considered more deeply by members of the public.

Malthus[9] stated that 'The tendency of all animal life is to increase beyond the nourishment provided for it'.

The reception of this idea for man is coloured by religious, social and political opinions, but there is no doubt that food deficiency and excessive numbers are factors in underdeveloped countries. However, in developed countries most people have enough to eat, and the problem arises from the consumption of excessive or ill-balanced diets. The known factors in diet for healthy living are within the common knowledge of most people. Increase in health giving food does not, however, necessarily result in improvement in health and the abolition of disease.

In the hospital service the doctors tend to treat disease. Clinical teaching is rightly focused on investigation, diagnosis, pathogenesis, clinical manifestations and treatment of disease. Questions which often receive insufficient attention are 'Why is the patient ill?.' 'How effective is the treatment and what risks are associated with it'?

What advice and care are needed by the patient or his relatives after completion of active measures?

Scientific approach to all disease processes is desirable, but practically the treatment may be related to environment outside hospital, and to resources within a hospital. In the latter there is a constant suggestion (or is it instruction) to assess the effectiveness of the expenditure in terms of money related to patients treated. This is the constant factor used to judge the National Health Service – the number of patients or bodies treated in relation to time and finance.

The B.M.J. of October 2nd[10] contains the first of 12 articles on 'Essentials of Health Economics'.

The patient is a body to be serviced and the disease process is to be eradicated as quickly as possible.

The assessment of the value of the Health Service is very difficult, and measurement of mortality and life expectancy does not take into account any fall in disability, pain, and suffering for any given life span. The treatment of osteo-arthritis of the hip is a good example of a condition which is not reflected in the evaluation of the contribution of the Health Service; relief of pain in this and other conditions is not measured in any recorded statistic. The quality of life is the consumer's interest and the statistics that are collected do not necessarily help to deal with this.

The state of the Health Service is too closely related to the reform of Society. There may be a danger of ignoring the achievements of the Health Service in pursuit of the vision of a transformed society. Professor Rudolf Klein[11] (Listener) states that 'to use the stonework of the N.H.S. as the building material for a new society is to risk breaking up what remains with all its imperfections, a formidable monument of social imagination, without any certainty that the new structure will ever get beyond the planning stage of rhetoric'.

Mr. Kennedy in the Reith Lectures in 1980[12] calls for more power for the consumer and restriction of the role of the State. There is to be a revolt against professionalism and bureaucracy and a development of a situation in which there is more power for the people. The N.H.S. is being brought into the political arena on the one hand and into the scientific field on the other. Are we to assume that the consumer will understand the way out of this situation?

We must not be surprised if the public, having listened to the arguments of the experts, decide to follow a reasonable and easily available line of research to find treatment. The consumer is used to shopping around for most things and is also biased towards challenging the professional view. If the situation is not an emergency one then the attitude of a rational shopper may take over and the Health Centre with its fixed medical vision may not be so attractive as the planners of health care may imagine. We must accept that there is a large element of uncertainty in the whole situation because there are so many unknowns which have not been tested or resolved.

In the service the consumer may be more critical of the style of the health care rather than the quality of service. Most people are locked into the Health Service, and their only way of breaking out is to escape into private or independent care or to fringe medicines of which there are many. The consumers wish to enjoy a competitive market in which they can impose their priorities and preferences rather than having the priorities and preferences of the provider-dominated N.H.S. system which is imposed upon them. Two factors stand out in all this, firstly communication and secondly freedom of choice.

If we agree that medicine is both an art and a science in old-fashioned terminology, or the application of the exact sciences and an understanding of the human personality in more modern terms, then any medical transaction between doctor and patient will be of a double nature.

This duality of approach to the patient has very direct consequences for the doctor-patient relationship right from the first contact. If the approach follows a scientific model the patient becomes an object. In other words the doctor sees not a sick person but a person with a disease. This disease becomes the object of scientific investigation. If, on the other hand, the doctor wants to establish a valid inter-personal relationship with a sick person, he will adopt an approach such that the patient communicates to him all his feelings at every level.

The choice of the doctor's attitude, scientific or personalised, is nearly always made at the start of the interview. If this choice, which determines the doctor's approach to his patient, fulfils the latter's requirements, then the transaction will take a favourable turn and give satisfaction to both of them.

Unfortunately, the doctor often fails to make a conscious decision based on his information and its evaluation, but follows a stereotyped model he has adopted during his education. His behaviour may become rigid and repetitive, and he cannot adjust his attitude to other neglected dimensions of the doctor/patient relationship.

This can happen both with those who use a technical and scientific model and those who use the psycho-analytical model of listening to the patient's emotional out-pourings.

An unconscious attitude is at work from the start of the doctor/patient relationship because of the fantasy world of both doctor and patient.

If to satisfy his job satisfaction and work content, the doctor needs 'good cases', then he is often going to be very disappointed. Often a patient will offer a comparatively trivial symptom and then start to talk about his inner conflicts.

This is a dangerous situation. The doctor may be so 'put off' that he may either through an emotional state or because he is put off his stroke, fail to get back to a regime which will help to diagnose an organic lesion. The patient may be so overjoyed to find that he is allowed to pursue his contained emotions that he will depart further and further from the salient clinical facts that he thinks are trivial and will not be helpful to the doctor.

Many of the 'patient diagnosis' may be right and are based on intuition and repeated family opinions. The doctor/patient relationship may be satisfactory at this level, but it is not based on science.

Thus, there are three types of consultation –

(1) The relation of the doctor as technician or scientist to a disease which he is going to study.

(2) The relation of the psycho-analyst who for technical reasons probes the emotional and conflictual life and allows himself to study the relationships which the patient has established and is trying to establish at that time.

(3) The relation of the general practitioner who does try to use both the above approaches as required. This ideal situation can lead to the largest number of satisfactory relationships.

A specialist must often tend to use the scientific approach. In fact, the patient may expect this. He is led to believe that a diagnosis must be supported by scientific tests and numerous investigations. A diagnosis made on a thorough clinical examination alone may lead to much discussion, and an up-to-date patient may quote articles or programmes on the media in order to try to goad the doctor into some extra activity.

The late Stephen Merivale used to say very frequently that consultants were very expensive, not because of their salaries but because of the workload that they inevitably generated. This is a fact that defeats the financial system in the Service, and it is a very naive person who thinks that senior doctors do not need adequate supportive facilities both for diagnosis and for interest sake. Life would be very dull indeed if the senior doctor was not allowed to follow his own personal interest, even in common and well established conditions.

We are so preoccupied with the concept of physical diseases and their causes that the Doctor's administration and unusual investigations may lead to both neurosis and anxiety. In fairness to the medical profession, one must admit that there is such preoccupation with the failure to diagnose an organic lesion, the reverse situation is rarely considered.

The harm done by treating as an organic disease a condition without a physical basis is rarely considered important, and iatrogenic anxiety is the price that has to be paid for medico-legal and social relationship.

The remedy for this doctor-induced anxiety is not easy, but it is made worse by faulty or absent communication and inconsistent actions.

Prevention includes a constant awareness of the side effects of the doctor-patient encounter, discrimination in choice of tests and in communication of the results, and as optimistic an attitude as possible. There are basically two main groups of patients— those striving for some sort of medical activity, and those dependent on the doctor's attitude. This leads us back to the original contrast between both scientific and psycho-analytical approaches, and no matter what advances may be made in medical matters the patient must be accorded time to develop a satisfactory approach. The doctor must take time to recognise the needs of his patient, and it may be that in group practices there should always be an attempt to develop special interests and skills as far as individuals in the group are concerned. This would be comparatively easy if there is time available. Perhaps the reduction of lists or the greater use of auxiliaries and nursing helpers might enable this development to take place.

In December 1981 in the Proceedings of the Royal Society of Medicine, Dr. Tudor Hart wrote about 'A New Kind of Doctor'.[13]

He stated that we are only beginning to define and learn the skills needed to help out patients to modify their behaviour.

Obviously, like any other effective procedure this cannot be done without time and resources, but the principal obstacle to progress has been our uncritical priority for salvage, however costly or unlikely to succeed, combined with refusal to accept more than token responsibility for either personalised or group patient education.

'Neither General Practitioners nor specialists are making and maintaining effective contact with the population at risk.

If effective contact is not made with the population, all strategies either remain an obstruction or become dictatorial benevolence.

May I read you extracts from an address to medical students by the Dean of the Faculty in a London Hospital.[14]

> 'You are preparing for a profession in which learning must continue as life lasts; in which self instruction and self reliance are absolutely essential. Therefore, cultivate them from the very beginning, for as Herbert Spencer has said "It is not knowledge stored up as intellectual fat which is of value, but that which has been converted into intellectual muscle".'
>
> 'The power of really seeing what we look at is perhaps one of the rarest gifts.
>
> From the very beginning of any public career one must school one's self to look upon nervousness as a disability which one must do one's utmost to conquer!!
>
> The two chief sources of mistakes are ignorance and carelessness. If you constantly avoid the latter the former will, to a large extent, take care of itself.
>
> It is inevitable in the early part of hospital life that one should class patients according to their diseases in order to acquire the necessary knowledge of types. If you are to be as useful as you can be however, you must remember that you will have to treat the individual and not a type, and that wide knowledge, only to be obtained by real study of human nature, will enable you to do this in the best possible manner.
>
> It is sometimes said that a scientific student does not make a good doctor. That can only be the case if relying on his pure science he insists on looking on the disease as it were through a microscope, magnifying the morbid portion and never seeing the human at all!'

This address was given in 1900 by Dame Louisa Aldrich Blake, who was Dean at the Royal Free Hospital. What foresight and how ageless the precepts. She would not be drawn into comparing women and men in the work which she was doing. As she said 'If you are good at your work you are certain to succeed, and if you are not you are certain to fail'. She said that you cannot have two standards of efficiency – the male and the female – and she avoided any suggestion of competition. The real point is to concentrate on your work and learn to do your job.

She was not content to rest on her laurels at any stage. A friend said of her at that time 'work was all absorbing and seemed no burden to her. I cannot think that she ever had to force herself like ordinary students. She seemed to have no desire for amusement'. She was the first women to hold the post of Registrar at the Royal Free Hospital from 1896–1908. She decided to become a general surgeon and succeeded in her aim.

From 1919–1925 she was a Consulting Surgeon at the Royal Free Hospital. At the age of 40 she took a course in accountancy and finance. She put the finances of the Medical School to right, and she was of great service to the Medical Women's Federation to which she became Honorary Treasurer in 1917.

Doctors may have to watch the gradual erosion of their authority and respect, because so many outside pseudo-scientific bodies are tending to suggest to the public that there is a scientific basis for most ailments, and both diagnosis and treatment are now available for all. In the N.H.S. the patient will expect the doctor to discharge his contractual obligation to him which is what he rightly considers to be his due.

Just because illness is associated with objective facts it appears that illness is those facts, that illness is a thing. Mr. Kennedy however, argued that illness is not a thing its a judgmental term.[15] Being ill in many cases is not a state but a status to be granted or withheld by those who have the power to do so—the doctors.

Status denotes a particular position in society assumed only after satisfying others that certain conditions have been observed.

We have opted for what Illich has called the medicalisation of life, the conversion of social and political ills into illnesses.

The W.H.O. definition of health is that 'Health is a positive condition – not merely the absence of disease but the total physical, mental and social well being'.

A healthy person is, in fact, usually unaware of his body, and perhaps we need more courses for lay people and fewer for the professionals. Health cannot be given to us by any one profession but we can be guided in our style of living.

Overall medicine is as it has always been – not a science but an art. Science may help, but it must not be allowed to rule the art.

Both orthodox and unorthodox medicine have plenty of failings. We can do with all the good that we can get from both. We cannot afford to waste any. This is why although orthodox medicine is free in the United Kingdom, many people turn to alternative medicines for which they have to pay. The history of human progress is full of people using phenomena that the establishment claims are not there.

Many people cannot see things that are different or 'odd'. These people are 'odd blind'. They cannot accept anything for which there is no scientific evidence.

At long last there is a hope that all kinds of medicine which only continue to exist because they work, can accept the useful disciplines of both unorthodoxy and orthodoxy into the far greater whole for the benefit of all.

### A FINAL QUOTATION

'We trained hard, but it seemed that every time we were beginning to form up into teams we would be re-organised. I was to learn late in life that we tend to meet any new situation by reorganising and a wonderful method it can be for creating the illusion of progress while producing confusion, inefficiency and demoralisation.'

Gaius Petronius. A.D. 66

Perhaps this is an unfair remark. We have both orthodox and independent practices and practitioners. We can only succeed in reducing the need for medical attention when the public return to positive healthy living and the doctors try to devise means of establishing better communication with the consumer or patient. Fringe medicine has to pick up the crumbs from under the rich man's table – let us reduce their burden by more tidy and healthy living.

### REFERENCES

1. MUNRO SMITH, 1917, History of Bristol Royal Infirmary. Arrowsmith, Chapter III Page 23.
2. Ib. Chapter XXI Page 279.
3. Ib. Chapter XXI Page 279.
4. Ib. Chapter XXIX Page 387.
5. In the Service of Medicine. Christian Medical Fellowship Leaflet No. 61, 1970.
6. Health Trends February 1982. No. 1. Vol. 14.
7. PICKERING G. 1977. Proceedings of the Royal Society of Medicine. Vol. 70. 16–20.
8. McKEOWN, THOMAS. 1979. The Role of Medicine. Basil Blackwell, Oxford.
9. MALTHUS. 1798. Essay on Population.
10. MOONEY, G. H., DRUMMOND, M. F. 1982. Essentials of Health Economics. B.M.J. Vol. 285. Page 949.
11. KLEIN, RUDOLF. 1980. The Listener.
12. KENNEDY, N. F. Reith Lectures 1980. The Listener 1980.
13. HART, T. Proc. of Royal Society of Medicine, December 1981. Volume 74 November 12.
14. RIDDELL, Dame Louisa Aldrich-Blake. Hodder & Stoughton Ltd.
15. KENNEDY, N. F. 1980. Reith Lectures. The Listener.

